# Acute stress responses of autonomous nervous system, HPA axis, and inflammatory system in posttraumatic stress disorder

**DOI:** 10.1038/s41398-023-02331-7

**Published:** 2023-02-03

**Authors:** Kristin von Majewski, Olga Kraus, Cosima Rhein, Marietta Lieb, Yesim Erim, Nicolas Rohleder

**Affiliations:** 1 Chair of Health Psychology, Institute of Psychology, Friedrich-Alexander Universität Erlangen-Nürnberg (FAU), Erlangen, Germany; 2 Department of Psychosomatic Medicine and Psychotherapy, Friedrich-Alexander Universität Erlangen-Nürnberg (FAU), Erlangen, Germany

**Keywords:** Depression, Predictive markers

## Abstract

Posttraumatic stress disorder (PTSD) does not only have direct consequences for well-being, but it also comes with a significant risk for severe somatic health consequences. A number of previous studies have pointed to alterations in stress systems in traumatized persons, as well as the inflammatory system, which might be important links in the pathway between trauma, PTSD, and health consequences. The aim of this study was to investigate acute stress responses in PTSD patients compared with healthy controls. Twenty-seven PTSD patients and 15 controls were exposed to the Trier Social Stress Test (TSST), and we measured salivary cortisol, salivary alpha-amylase (sAA), plasma interleukin-6 (IL-6), as well as heart rate and heart rate variability (HRV) at different time points before, during and after the stress test. Results revealed similar stress responses between patients and controls, but lower baseline cortisol levels and higher IL-6 baseline levels in PTSD patients. Increases in sAA stress responses were significantly lower in patients, while sAA concentrations were higher in the PTSD group during intervention. HRV was markedly decreased in patients and showed a significantly blunted acute stress response with a slower recovery after TSST. These results confirm previous findings of marked stress system dysregulations in PTSD and add to the literature on acute stress reactivity in PTSD which appears to show stress system-specific changes. Overall, these results have implications for our understanding of potential risk and resilience factors in the response to trauma.

## Introduction

Traumata play an ever-increasing role in human life, for example as a consequence of military conflicts and natural disasters, and related phenomena such as migration or flight, or through human behavior in personal inter-individual conflicts. Possible consequences after a traumatic event are posttraumatic stress disorder (PTSD) and trauma-associated disorders with a prevalence of 7–12% [1]. In addition to psychological suffering, PTSD is not only associated with comorbid psychiatric or somatic disorders, but also with higher mortality from heart disease and a number of alterations in the function of stress systems, as well as in further dependent or related homeostatic systems [2]. A common characteristic of somatic diseases or physical symptoms comorbid with PTSD is that all of those are related with, or in many cases, stimulated by exaggerated chronic inflammation [3].

Several current reviews provide an excellent summary of the role of inflammation in the etiology of the most relevant chronic diseases of the industrialized world [3]. In one of these reviews, despite inconsistent findings, Sumner et al. [4] report results indicating that inflammation increases susceptibility to PTSD after trauma or that PTSD can lead to altered inflammatory processes, suggesting that the relationship may be bidirectional. It has therefore been suggested that a possible mechanism linking PTSD with these conditions is dysregulated stress systems, ultimately leading to the disinhibition of inflammatory processes [5–9].

Most notable and best described are alterations of the hypothalamus-pituitary-adrenal (HPA) axis and the autonomic nervous system (ANS). Although results are not consistent across all studies, considering relevant factors such as comorbidities and daily profiles, the current data indicate a dysregulated HPA axis system characterized by hypocortisolism and enhanced feedback sensitivity in patients with PTSD [10, 11].

In addition to the HPA axis, alterations have also been found in the ANS, mainly in the sympathetic component (SNS). A consistent finding of increased norepinephrine secretion in patients with PTSD is noticeable between studies [11, 12]. However, there is an inconsistency in referring to other alterations such as dopamine or epinephrine [13–15].

A further marker for SNS activity and reactivity is salivary alpha-amylase (sAA). Thoma et al. [16] for example, found an increased sAA awakening response in PTSD and altered diurnal sAA rhythm compared with controls. This is in line with Vigil et al. [17], who found a higher sAA activity by taking two saliva samples prior to and subsequent to questionnaires 60–90 minutes apart in survivors of hurricane Katrina compared with controls. Keeshin et al. [18], however, reported no differences in diurnal sAA secretion between PTSD patients and controls, but an association between PTSD symptoms and higher overall morning levels of sAA. Additional results with sAA also point to exaggerated stress reactivity in traumatized individuals and/or PTSD [19].

Much less is known about acute stress reactivity, although evidence seems to support the notion that acute cortisol responses to experiencing a traumatic event are associated with the development of trauma-related psychopathology. Ehring et al. [20] reported that lower levels of salivary cortisol predicted greater symptom levels of PTSD in accident survivors six months later. Delahanty et al. [21] found significantly lower urinary cortisol in motor vehicle accident victims measured immediately after the accident predicted PTSD development. These findings not only highlight the importance of being able to mount a stress response in the direct aftermath of trauma, but also are further in line with studies showing that pre-trauma stress system status, including stress reactivity, can be predictors of PTSD development after experiencing trauma [22].

Findings showing that pre- and peri-trauma stress reactivity appears to be predictive of PTSD development, raise the question whether stress reactivity measured in individuals with chronic PTSD could predict symptom maintenance, exacerbation, and/or success of therapeutic interventions. However, only few studies have addressed the role of acute stress reactivity in PTSD. Simeon et al. [23] for example reported no differences between cortisol stress reactivity in acute stress experiences in PTSD patients compared to healthy controls but a trend towards blunted cortisol stress reactivity in the PTSD group with comorbid dissociations. Roelofs et al. [24] compared patients with social anxiety disorder with PTSD and a healthy control group exposed to the Trier Social Stress Test (TSST) and did not find differences in cortisol responses. In contrast, Zaba et al. [25] reported significantly blunted cortisol responses in PTSD patients compared to a healthy control group. While there are only few studies on acute cortisol reactivity in a laboratory stress situation, the overall tendency appears to be that cortisol responses are blunted in PTSD patients in the laboratory. In light of the importance of inflammatory processes for somatic consequences of PTSD, and inflammation’s effects on the CNS, which could have implications for symptom maintenance and exacerbation [26], and which could negatively interfere with treatment success, it is surprising that almost nothing is known about inflammatory stress responses. Only one study by Renner et al. [27] found no elevated IL-6 levels but an increase in anti-inflammatory IL-10 during TSST in PTSD patients compared to healthy controls.

In the present study, we, therefore, set out to investigate acute stress responses in PTSD patients compared with healthy controls, and focusing on inflammatory acute stress responses, as well as the hypothesized upstream regulators of inflammation, i.e., HPA axis and SNS stress responses.

## Material and methods

### Participants

Participants were *n* = 27 trauma-exposed persons (4 male) clinically diagnosed with PTSD according to ICD-10 with an average age of *M* = 38.48 (SD = 12.62), as well as 15 (1 male) healthy controls (age *M* = 29.67; SD = 9.48). Exclusion criteria for control participants were experience with the TSST, and any psychiatric disorder, pregnancy, or the use of immunosuppressive drugs at the time of study participation. In addition, control participants completed the ETI [28] to exclude the presence of PTSD. At time of enrollment, patients were being treated in the day clinic of the Psychosomatic Medicine and Psychotherapy´s Department at the University Hospital Erlangen. Four participants had to be excluded from data analyses due to high baseline values of cortisol or IL-6 (three patients) or missing data (one patient from PTSD group). Three participants were excluded due to cortisol baselines above 10 nmol/l (two patients, one healthy control), because high baselines are generally presumed to prevent acute stress responses due to negative feedback mechanisms (e.g., [29]). The resulting final sample size for analysis of cortisol responses was *n* = 38 (24 PTSD, 14 healthy controls). Three participants had to be excluded from the IL-6 sample, two of those because of baseline IL-6 concentrations above 5 pg/ml, which is generally indicative of acute infection or other inflammatory processes [29], and one due to missing data caused by laboratory error (all three from the group of patients; these were the same individuals also excluded for cortisol analyses). The resulting final sample size for analyses of inflammatory responses was *n* = 39 (24 PTSD, 15 healthy controls).

Inclusion criteria were an age between 18 and 65 years as well as a diagnosis of PTSD according to ICD-10. Exclusion criteria were pregnancy, breastfeeding, acute psychosis, acute suicidality, current substance abuse, a BMI < 17 kg/m^2^, or current contact to the offender related to the traumatic event. Twenty PTSD patients and one control met the criteria of comorbid depression (mild *n* = 2, moderate *n* = 3, severe *n* = 15). Furthermore, two patients were diagnosed with somatic symptom disorder, five patients with anxiety disorder (of these two individuals with panic disorder, one person with agoraphobia, two individuals with social phobia), one patient with borderline-personality disorder, and one person with bulimia nervosa. Patients were enrolled during their first week of an 8-week psychotherapy treatment. Diagnostic interviews and psychometric assessments were conducted one week before the laboratory assessment. Four patients received anti-inflammatory and 16 patients were treated with anti-depressive drugs (mainly SSRIs or SNRIs) during the observation period. Moreover, seven patients used hormonal contraceptives, five patients reported taking thyroid medication and one person was medicated with atypical neuroleptics. Additionally, psychotropic drugs were used in the course of therapy, if needed. The study protocol was approved by the Ethics Committee of the Friedrich-Alexander-University Erlangen-Nuremberg and all participants were asked to give written informed consent prior to participation.

### Procedure

Patients were asked to complete a diagnostical assessment one week after admission to the day clinic. Experienced clinicians (Y.E., M.L., as well as colleagues from the Department of Psychosomatic Medicine and Psychotherapy at FAU directed by Y.E.) performed psychometric assessments of trauma-related symptoms (measured by Essen Trauma Inventory, see 3.3.1) and the most common comorbid mental disorders of PTSD. Laboratory sessions took place within the following two weeks, in the afternoon, to control for circadian variation of biological parameters. During the laboratory session, an indwelling venous catheter was placed into the non-dominant forearm for blood collection. After this, a 30-minute resting period followed to recover from the potential stress response to venipuncture. After that, a first blood sample was taken, and the TSST (described below) was administered, followed by additional samples one, 10, 30, and 90 minutes post-TSST. All blood samples were taken into EDTA tubes (Sarstedt, Nümbrecht, Germany), stored on ice for a maximum of ten minutes, and then centrifuged and aliquoted as described below. Saliva samples were taken into cortisol (blue top) salivette devices (Sarstedt) before, as well as one, 10, 20, 30, 45, 60, and 90 minutes post-TSST for later assessment of salivary cortisol and amylase. After the last sample, participants were debriefed and allowed to leave the laboratory. Heart rate was recorded using commercial exercise watches (V800, Polar Electro Oy, Kempele, Finland) and chest strap (H7, Polar). We analyzed eight 5-minute-intervals starting at −30, −10, 0, +5, +10, +20, +30, +60 minutes, relative to the start of the TSST (three of these correspond to specific segments during TSST, i.e., preparation [0 min], speech [+5 min], arithmetics [+10 min]).

To induce acute psychosocial stress, we subjected all participants to the standardized laboratory stress paradigm TSST [30]. The TSST is considered the gold standard for acute stress induction in the laboratory and was demonstrated to induce the most robust cortisol responses [31]. The procedure was divided into three parts: a 3-minute preparation period, a 5-minute speech (with the topic “how personality qualifies the subject for a job”), and a 5-minute arithmetic task (backward arithmetic of 2043 in steps 17). The TSST was performed by two jury members with neutral facial expressions. Participants were informed that the judges were trained in analyzing verbal and non-verbal behavior. Speech and arithmetic parts of the TSST were videotaped.

The study design was approved by the Ethics Committee of the Friedrich-Alexander-University Erlangen-Nürnberg (FAU, ID 340_16B) and conducted in concordance with the Declaration of Helsinki.

### Measures

#### Psychometric assessment

All trauma-exposed participants completed the Essen Trauma Inventory (ETI) [28]. The ETI is used to diagnose trauma-related disorders and assesses potentially traumatic experiences or threats to life, particularly regarding the four core symptoms intrusion, hyperarousal, avoidance, and dissociation. The inventory was used in the long version as a self-assessment questionnaire (46 items). To assess psychological stress responses, we administered the Primary Appraisal Secondary Appraisal Questionnaire (PASA) [32], to measure threat and challenge perceptions during the TSST, and the Thoughts Questionnaire (TQ) [33] to measure post-stress rumination.

#### HPA axis reactivity

To assess HPA axis reactivity, we measured salivary-free cortisol from samples collected at the time points indicated above. All salivettes were centrifuged and samples were aliquoted and stored at -20 °C until processing. Cortisol was measured in duplicate using a competitive chemiluminescence immunoassay (IBL-International, Hamburg, Germany). Inter- and intra-assay coefficients were below 10%.

#### Autonomic nervous system reactivity

To quantify ANS stress responses we used two different measures to obtain a more complete picture. Heart Rate Variability (HRV) was extracted from continuous beat-to-beat recordings exported from Polar V800 and H7 monitors (Polar, see above). The software Kubios HRV (Universität Kuopio, Finnland, Version 2.0) was used to compute HRV parameters based on recorded interbeat (R-R) intervals. We used the root mean square of successive differences between normal heartbeats (RMSSD) as an index for vagal tone [34]. RMSSD was expressed as ms^2^ and log-transformed to achieve normal distribution. RMSSD and heart rate (HR) were calculated for eight 5-min intervals. These intervals started at −30, −10, 0, +5, +10, +20, +30, +60 minutes, relative to the start of the TSST (intervals 3, 4, and 5 were taken during the TSST).

We further measured sAA from the same saliva samples taken for cortisol using an in-house procedure, using reagents from DiaSys Diagnostic Systems GmbH (Holzheim, Germany). In brief, saliva was diluted at 1:625 with ultrapure water, and diluted saliva was incubated with substrate reagent (a-amylase CC FS; DiaSys Diagnostic Systems) at 37 °C for three minutes before a first absorbance reading was taken at 405 nm with a Tecan Infinite 200 PRO reader (Tecan, Crailsheim, Germany). A second reading was taken after five minutes of incubation at 37 °C and the increase in absorbance was transformed to sAA concentrations (U/ml), using a standard curve prepared using “Calibrator f.a.s.” solution (Roche Diagnostics, Mannheim). Intra- and inter-assay coefficients of variation were below 10%.

#### Plasma inflammatory reactivity

To quantify low-grade systemic inflammation, we measured interleukin-6 (IL-6) in EDTA blood samples obtained at five-time points (see above). Blood samples were centrifuged and plasma was aliquoted and stored at −80 °C until batch processing at the end of data collection. IL-6 concentrations were determined using a commercial high-sensitivity ELISA (Quantikine HS; R&D Systems, Minneapolis, MN, USA), with a lower limit of detection of 0.09 pg/ml. Inter- and intra-assay coefficients were below 10%.

### Statistical analyses

All statistical analyses were performed using JASP (Version 0.12; JASP Team, 2020). Kolmogorov–Smirnov tests were computed prior to statistical analyses to test for normal distribution as well as homogeneity of variance of all dependent variables. When logarithmic transformations were necessary, variables were transformed by applying the formula *x*_ln_ = ln(*x* + 1). Greenhouse–Geisser corrections for repeated measures were calculated where appropriate (indicated by decimal degrees of freedom values). Data were expressed as means ± SEM, and *p* < .05 was set as the criterion for significance. To test for stress-induced changes in cortisol and IL-6, we used *t*-tests and analysis of variance (ANOVA) for repeated measures, with the within-subject factor “Time” (five-time points for IL-6, and eight-time points for cortisol) and the between-subjects factor “Group” (experimental group vs. control group). For cortisol, we additionally computed the area under the curve with respect to baseline (AUCg) according to the formula suggested by [35]. For all parameters, we also calculated the relative increase with respect to the baseline (i.e., the baseline level of each parameter was subtracted from the post-stress level), or the maximum increase (max increase), as additional indices of stress-induced changes. To calculate the max increase, the baseline level of each parameter was subtracted from the peak level. Similar analyses were performed for heart rate (variability) parameters. In addition, we performed exploratory analyses of bivariate associations between stress system baseline levels and stress responses using Pearson correlations.

## Results

### Psychological stress responses

We found no group differences in situational cognitive assessments regarding primary and secondary appraisal during the TSST (PASA questionnaire: *t* (31) = 1.89, *p* = 0.07), but significantly higher automatic negative thoughts after the TSST in patients vs. controls (TQ: *t* (36) = 4.25, *p* < 0.001).

### HPA axis reactivity

**Fig. 1:**
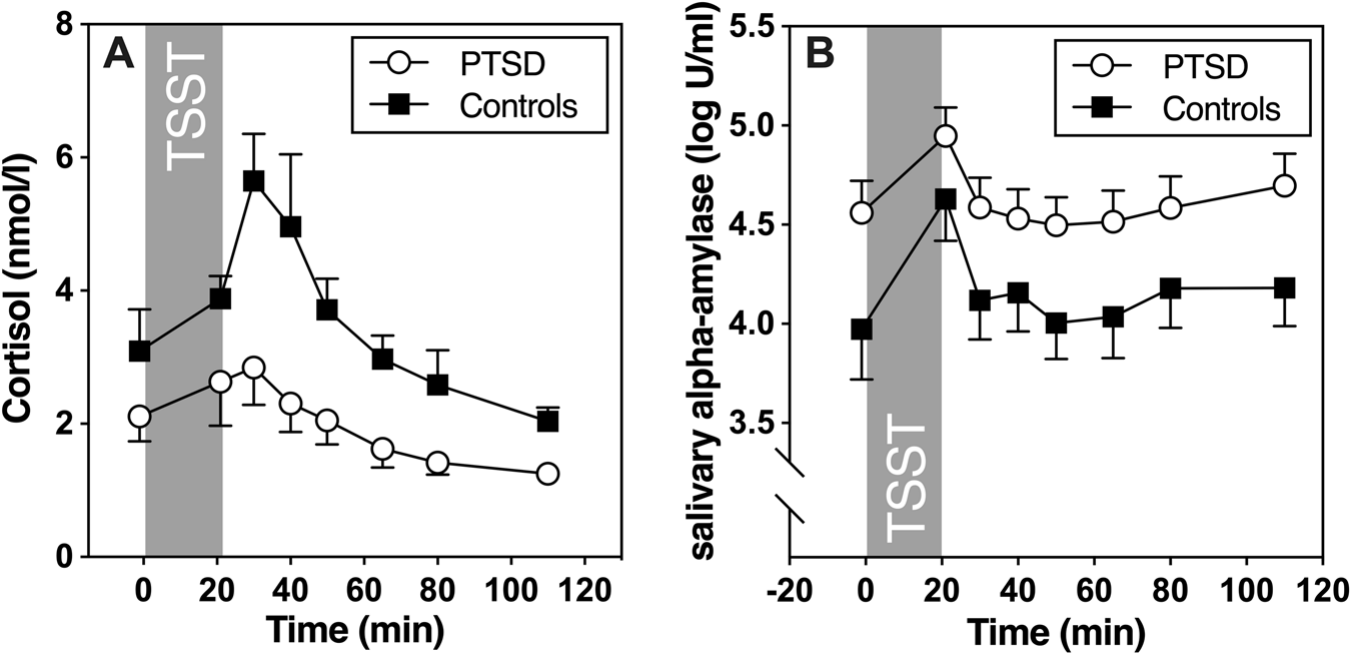
HPA axis and salivary alpha-amylase reactivity to acute psychosocial stress. **a** Salivary free cortisol and **b** salivary alpha-amylase (sAA) concentrations before and after TSST. Figure shows mean and SEM.

We first examined whether TSST exposure induced increases in salivary cortisol using repeated measures ANOVA. PTSD patients showed significantly lower baseline cortisol than healthy controls (*F* (36, 1) = 9.36; *p* = .004). A significant time effect (*F* (3.81, 137.12) = 23.03; *p* = <0.001) indicates a cortisol response during intervention, which did not differ between groups (group by time interaction: *F* (3.81, 137.12) = 1.53, *p* = 0.20; see Fig. 1A). The general observation of generally lower cortisol levels during course of the study, without differences in reactivity, was confirmed by significantly lower AUCg (*t* (36) = −3.07, *p* = .004) in the PTSD group, with similar maximum cortisol increase values (*t* (15) = −0.79, *p* = 0.44).

### Autonomic nervous system reactivity

Baseline values for sAA were significantly higher in PTSD patients (*t* (39) = 4.20, *p* = 0.047). Psychosocial stress-induced increases in sAA, which were found in both groups (time effect: *F* (5.12, 199.49) = 15.88; *p* = <0.001), with marginally higher amylase levels in PTSD patients (group effect: *F* (39, 1) = 3.55; *p* = 0.07). Increases in sAA, however, were significantly lower in PTSD patients compared to controls (*t* (39) = −2.34, *p* = 0.024; See Fig. 1b).

**Fig. 2:**
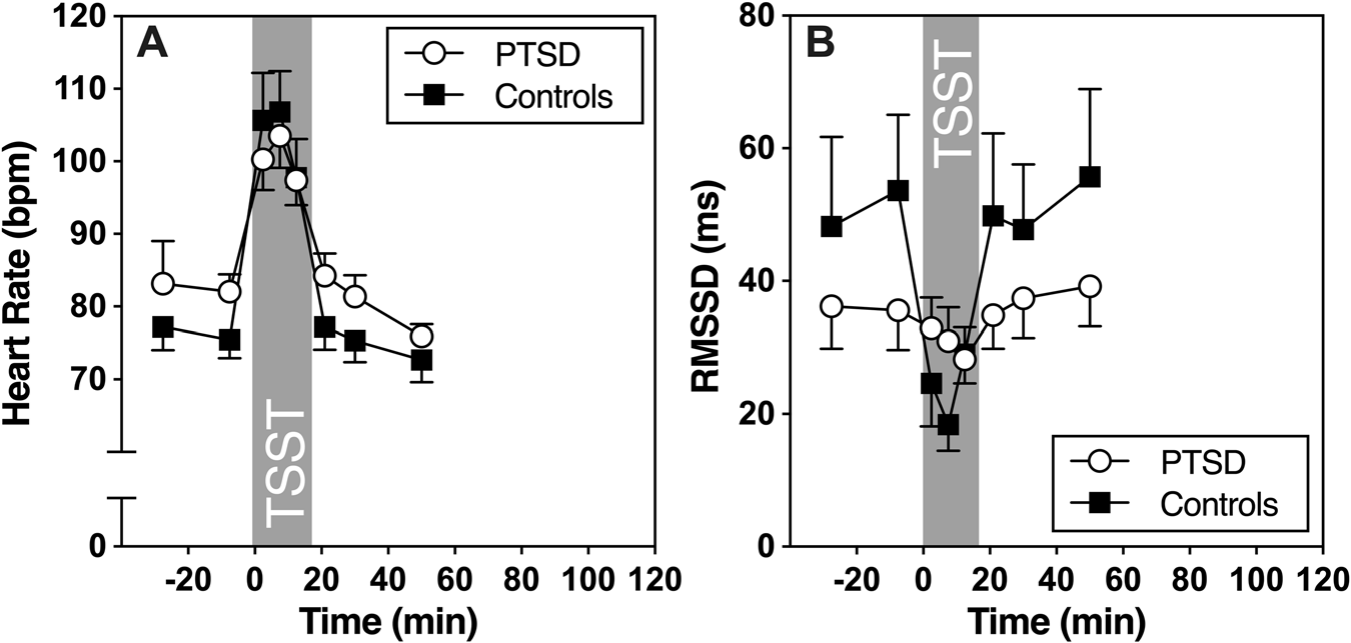
Heart rate and heart rate variability reactivity to acute psychosocial stress. **a** Heart rate (HR) and **b** heart rate variability (HRV; measured as RMSSD) before and after TSST. Data points represent bins of 5-min intervals. Figure shows mean and SEM.

With regard to electrophysiological markers of ANS (re-) activity, we analyzed stress responses of heart rate (HR) and RMSSD as an index of HR variability (HRV). For HR, repeated measures ANOVA revealed a significant time effect (*F* (1.91, 61.19) = 22.01; *p* < 0.001) indicating higher stress-induced changes in HR. However, no group differences in overall HR (group effect: *F* (32, 1) = 0.36, *p* = 0.56), and in HR stress responses were observed (group by time interaction: *F* (1.91, 61.19) = 0.81; *p* = 0.45; See Fig. 2a). For RMSSD, repeated measures ANOVA revealed a significant time effect (*F* (2.85, 91.30) = 12.60; *p* < 0.001) indicating stress reactivity. Furthermore, a significant group-by-time interaction revealed group differences in HRV stress responses over time (*F* (2.85, 91.30) = 5.59, *p* < 0.001). As shown in Fig. 2b, healthy participants showed a significant decrease in RMSSD in three intervals during the TSST compared to baseline, and a fast recovery thereafter. This pattern was absent in PTSD patients, who showed a generally lower RMSSD without stress reactivity (*F* (1, 32) = 0.28, *p* = 0.60).

### Plasma inflammatory reactivity

**Fig. 3:**
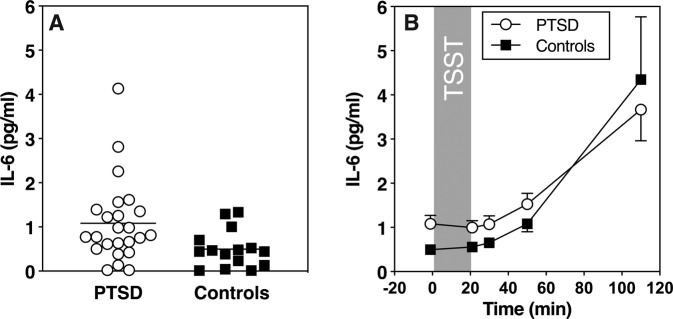
Plasma inflammatory activity and reactivity to acute psychosocial stress. **a** Interleukin-6 (IL-6) baseline and **b** stress response to TSST. **a** Shows individual data points and mean, **b** shows mean and SEM.

To test for group differences in IL-6 stress responses, a further repeated measures ANOVA was computed. Results showed a significant time effect (*F* (1.38, 51.16) = 41.83, *p* = <0.001), indicating stress-induced increases in all participants, but no significant group effect (*F* (1, 37) = 1.98, *p* = 0.17) or group by time interaction (*F* (1.38, 52.16) = 1.25, *p* = 0.29; see Fig. 3b). We also did not find significant group differences regarding IL-6 reactivity (*t* (37) = −1.33, *p* = 0.19). However, baseline IL-6 values were significantly higher in PTSD patients (*t* (37) = 2.51, *p* = 0.017; see Fig. 3a).

### Exploratory analyses of associations between stress responses

We exploratively tested for associations between baseline levels and stress of all stress systems using bivariate Pearson correlations separately for patients and controls. We did not find any associations except for the correlation of salivary Cortisol AUCg and IL-6 response (*r* = −0.41, *p* = 0.048) in the patient group. Due to sample size limitations, we did not test for further interactions between stress systems.

## Discussion

In this study, we tested whether there was a difference in acute stress response in patients with posttraumatic stress disorder compared to a healthy control group. We aimed to add a further contribution to this research topic, as there are few studies on acute cortisol reactivity in PTSD, and even fewer about inflammatory stress responses in this group of patients. With regard to HPA axis reactivity, results were only partially in line with our expectations, as stress reactivity did not differ between patients and controls. Instead, results revealed similar stress responses, but lower baseline cortisol levels and lower AUC in PTSD patients throughout TSST. Amylase baseline values were higher in patients compared with controls, while overall amylase concentrations showed a trend to higher values in the PTSD group. Amylase stress responses were lower in PTSD relative to controls. Electrophysiological markers of ANS reactivity revealed a mixed pattern, with HR stress responses similar in patients and controls, but a striking absence of HRV stress reactivity in PTSD, together with generally lower HRV. Finally, plasma inflammatory reactivity showed no significant differences between patients and controls, but baseline IL-6 was significantly higher in the PTSD group.

Regarding overall cortisol in PTSD patients, where abundant literature is available [5, 36–38] our findings are consistent with these levels being lower in PTSD patients compared to a healthy control group. However, regarding cortisol reactivity to acute stress, where previous literature is still scarce and does not show a common pattern, our results did not reveal a significant group difference. This is in line with Simeon’s and Roelofs’ findings but in contrast to that of Zaba et al. [23–25]. Worth mentioning, Zaba et al. only investigated female patients in their study, which could be the first methodological reason for their results not being extendable to the general population. Perhaps even more remarkable is that Zaba et al.’s patients were treated in an outpatient clinic, whereas the patients in our study, as well as those in the study by Roelofs et al. and Simeon et al., had not yet received any therapy or were in its very early phase. Future research should hence investigate the differences in acute stress response between the same PTSD patients before, during, and after therapy. Interestingly, Zaba et al. [25] suggested two different HPA axis reactivity subgroups in PTSD, of which only one would show a blunted HPA-axis response, which greatly diminished the overall HPA axis response of the entire sample. This classification is based on the work of Miller et al. [39], who distinguished between cortisol responders and non-responders after 1.5 nmol/l or a 15.5% increase in cortisol levels in the TSST. They speculated that the HPA axis might influence symptoms not directly belonging to the PTSD core symptoms, but rather for example trauma-associated dissociative symptoms. This could potentially be explained by a higher rate of PTSD responders within the sample of those studies, such as ours. Furthermore, within the PTSD non-responder group, comparatively higher disease severity was found, accompanied by higher comorbidity with anxiety disorders, more negative interpretation of trauma symptoms, and significantly more trauma-associated dissociative symptoms. Literature suggests that a strong acute stress response immediately after trauma could lead to less severe symptoms of potential PTSD [20, 21]. In line with this reasoning, we assume that having a higher acute cortisol stress response could be postulated as a protective factor regarding PTSD severity. In this context, it is further interesting to note that lower HPA axis stress reactivity has been linked to increased susceptibility to inflammatory diseases [40]. To further investigate this relationship, we have, as a core part of our study, measured stress-induced inflammation, which will be discussed further below.

Considering sAA as an indicator of sympathetic and RMSSD for parasympathetic activity, we may interpret our result as an altered ANS reactivity in PTSD patients. As an example and in line with Keeshin et al. [18], we found higher sAA baseline values in PTSD. In addition, we found a trend towards significance with marginally higher overall amylase levels, together with significantly lower sAA increases in response to stress in PTSD patients. Yoon and Weierich [41] found higher amylase levels in traumatized patients in response to trauma reminders. A possible explanation for why their participants responded stronger than ours in this TSST study could be that sAA reactivity to trauma memories may have a greater sensitivity as a potential marker of hypervigilance in the absence of threat, which is a characteristic feature of trauma exposure, in contrast to the generally unpleasant situation of the TSST [41].

Our findings are in line with prior studies showing an alteration in the sympathovagal balance in PTSD patients [42–45]. Significant group differences with respect to HRV stress response were found, with generally lower RMSSD without stress reactivity, and slower recovery after TSST in PTSD patients. This implies an altered vagal activity and reactivity in PTSD patients, which might be a physiological consequence of a generally disturbed autonomic response to emotionally relevant stressors. This has several interesting implications. First, a meta-analysis suggested that decreased HRV could be considered an endophenotype associated with increased inflammation in PTSD [46]. Our results are able to support those findings as we also found increased baseline IL-6 values, which will be discussed below. Second, Carnevali et al. [47] concluded in their review that individuals under resting conditions who have higher vagally mediated HRV, perform better on resilience questionnaires, recover more efficiently from acute psychological stress, and are less susceptible to developing PTSD and depression-related symptoms. Considering this and investigating a PTSD group in our study, we might interpret the findings of our study that lower HRV is a higher risk for the development and severity of PTSD. Surprisingly, although Cohen et al. [42] described a significantly higher heart rate in the resting state in PTSD patients, we could not replicate these findings. This could be explained by the high number of patients taking selective serotonin reuptake inhibitors (SSRIs) at the time of testing, which has a mood stabilization and anxiety-reducing effect and can influence the activity of the sympathetic nervous system [48].

Inflammatory baseline values have also been found to be dysregulated in patients suffering from PTSD [49–52], which is supported by our findings of higher IL-6 baseline values in PTSD patients. In line with Renner et al. [27], we also found no significant differences in IL-6 responses to acute stress. However, they did find higher values of IL-10 while performing the TSST in PTSD patients. With respect to this, they postulated that acute stress could influence the immune response of PTSD patients in terms of increased levels of the anti-inflammatory IL-10 compared to healthy controls, rather than the pro-inflammatory IL-6 as found in spontaneous measurements. These are conceivable underlying mechanisms that may also have occurred in our study. However, the samples of Renner et al. [27], also showed high comorbidity rates such as 16 out of 17 PTSD patients also suffering from major depression. As Pace [53] found increased inflammatory responses regarding IL-6 to psychosocial stress (TSST) in depressed male patients with early life stress, but no relationship between Childhood Trauma Questionnaire scores and immune variables, the specific role of the immune system in acute stress in PTSD patients remains unclear. However, our results support the few findings of no differences in IL-6 acute stress responses in PTSD patients compared to controls. Further research is needed to clarify the effect of the prescribed medications, the large number of comorbidities especially the relationship with depression, lack of non-PTSD traumatized controls, and the relatively small sample size, leading to our limitations of the study which will be discussed below.

PTSD is a disease that can lead to reduced quality of life, effects on somatic health, and an increased risk of mortality. Understanding the pathways from PTSD experience to health consequences is therefore an important research question. With our study, we were able to make a further contribution regarding biological alterations in PTSD patients. Furthermore, we found changes in the ANS acute stress response, with diminished reactions in PTSD patients. In this context, it is important to conduct further research with respect to the acute stress response as well as progression during therapy. Longitudinal studies could investigate such persons’ development in terms of health.

The following limitations should be considered when interpreting our results. First, the series of IL-6 measurements included only five different points in time. Therefore, the functional predictors may not capture the full dynamic of the stress response. Future studies may therefore benefit from an expanded and possibly more detailed observation period. Second, our sample size was relatively small and the sample was largely female. A larger sample size as well as a more balanced sex distribution might have allowed us to detect more subtle relationships and more generalizable results. This would further allow a more detailed investigation of the potential moderating influence of sex on the effect of IL-6 reactivity and also enable the possibility for further analyses regarding interactions of the stress systems. Finally, a number of patients received medication and were diagnosed with comorbidities. This makes generalization difficult. Future studies should try to select patients without medication.

Taken together, our findings suggest that compared with controls, PTSD may lead to lower levels of cortisol and higher inflammation-related markers but no differences in acute stress response. These findings suggest lower cortisol as a factor regarding the severity of possible PTSD. Furthermore, we found dysregulations in SNS both in baseline values and acute stress reactions, which could indicate a risk factor for the development and progression of PTSD. Further research should investigate the relationship between therapy success and biomarkers in order to identify possible protective and risk factors.
